# Reduced hepatitis B and D viral entry using clinically applied drugs as novel inhibitors of the bile acid transporter NTCP

**DOI:** 10.1038/s41598-017-15338-0

**Published:** 2017-11-10

**Authors:** Joanne M. Donkers, Benno Zehnder, Gerard J. P. van Westen, Mark J. Kwakkenbos, Adriaan P. IJzerman, Ronald P. J. Oude Elferink, Ulrich Beuers, Stephan Urban, Stan F. J. van de Graaf

**Affiliations:** 1 Tytgat Institute for Liver and Intestinal Research, Amsterdam Gastroenterology and Metabolism, AMC, Amsterdam, The Netherlands; 2 Department of Infectious Diseases, Molecular Virology, University Hospital Heidelberg, Heidelberg, Germany; 3 Medicinal Chemistry, Leiden Academic Centre for Drug Research, Leiden University, Leiden, The Netherlands; 4 Aimm Therapeutics, Amsterdam, The Netherlands; 5 Department of Gastroenterology & Hepatology, Amsterdam Gastroenterology and Metabolism, AMC, Amsterdam, The Netherlands; 6 German Center for Infection Research, Heidelberg University, Heidelberg, Germany

## Abstract

The sodium taurocholate co-transporting polypeptide (NTCP, *SLC10A1*) is the main hepatic transporter of conjugated bile acids, and the entry receptor for hepatitis B virus (HBV) and hepatitis delta virus (HDV). Myrcludex B, a synthetic peptide mimicking the NTCP-binding domain of HBV, effectively blocks HBV and HDV infection. In addition, Myrcludex B inhibits NTCP-mediated bile acid uptake, suggesting that also other NTCP inhibitors could potentially be a novel treatment of HBV/HDV infection. This study aims to identify clinically-applied compounds intervening with NTCP-mediated bile acid transport and HBV/HDV infection. 1280 FDA/EMA-approved drugs were screened to identify compounds that reduce uptake of taurocholic acid and lower Myrcludex B-binding in U2OS cells stably expressing human NTCP. HBV/HDV viral entry inhibition was studied in HepaRG cells. The four most potent inhibitors of human NTCP were rosiglitazone (IC_50_ 5.1 µM), zafirlukast (IC_50_ 6.5 µM), TRIAC (IC_50_ 6.9 µM), and sulfasalazine (IC_50_ 9.6 µM). Chicago sky blue 6B (IC_50_ 7.1 µM) inhibited both NTCP and ASBT, a distinct though related bile acid transporter. Rosiglitazone, zafirlukast, TRIAC, sulfasalazine, and chicago sky blue 6B reduced HBV/HDV infection in HepaRG cells in a dose-dependent manner. Five out of 1280 clinically approved drugs were identified that inhibit NTCP-mediated bile acid uptake and HBV/HDV infection *in vitro*.

## Introduction

The sodium taurocholate co-transporting polypeptide (NTCP, *SLC10A1*) plays a pivotal role in the enterohepatic circulation of bile acids as the main uptake transporter of conjugated bile acids in the liver^1^. NTCP is exclusively expressed at the basolateral membrane of hepatocytes, where it extracts bile acids from the portal vein in a sodium-dependent manner. Recently, NTCP was identified as the entry receptor for the hepatitis B virus (HBV)^2,3^. Viral infection with Hepatitis B causes acute and chronic inflammation of the liver and is one of the major infectious diseases worldwide^4^. Chronic HBV infection is nowadays treated by interferon-alpha or nucleoside/nucleotide analogues but this rarely results in viral elimination. On top of HBV infection, it is estimated that 5% of HBV patients suffer from a co- or superinfection of the related hepatitis delta virus (HDV)^5–7^. This RNA virus propagates only in HBV infected cells as HBV-encoded envelope proteins are utilized for HDV envelopment. For HDV no antiviral treatment is available. Furthermore, recent data show that NTCP also plays a modulatory role in hepatitis C virus (HCV) host cell infection and cell to cell transmission by bile-acid-mediated regulation of antiviral responses of the innate immune system^8^.

HBV/HDV binding to the NTCP protein on the host cell is mediated by the myristoylated preS1-domain of the HBV envelope L-protein^9^. Myrcludex B, a synthetic peptide mimicking the HBV-specific NTCP-binding domain^10^, strongly interferes with viral docking and therefore abrogates HBV/HDV infection in established cell culture models of HBV/HDV infection^11,12^. Currently, Myrcludex B is being tested in phase II clinical trials as a novel means to inhibit HBV/HDV entry^13–16^. Largely reduced HDV RNA and HBV DNA serum levels were detected in a cohort of chronic hepatitis delta patients treated with Myrcludex B for 24 weeks^13^.

Upon NTCP inhibition by Myrcludex B, bile acid transport is affected^17^. However, whether NTCP inhibition can also affect drug exposure or clearance is unknown. Therefore we formulated the following three research questions. First, can we identify clinically applied and licenced drugs as novel NTCP inhibitors? Second, and in the context of drug-drug interaction, do such drugs compete with Myrcludex B for NTCP and vice versa? Third, can these novel NTCP inhibitors reduce HBV/HDV infection *in vitro*? In two screening approaches we evaluated 1280 clinically approved drugs on interference with NTCP-mediated bile acid transport and on occupation of the Myrcludex B binding site of NTCP. As a result, we describe the identification of 5 novel NTCP inhibitors with an estimated IC_50_ value below 10 µM that reduce HBV/HDV infection in HepaRG cells in a dose-dependent manner.

## Results

### Identification of novel NTCP inhibitors

**Figure 1:**
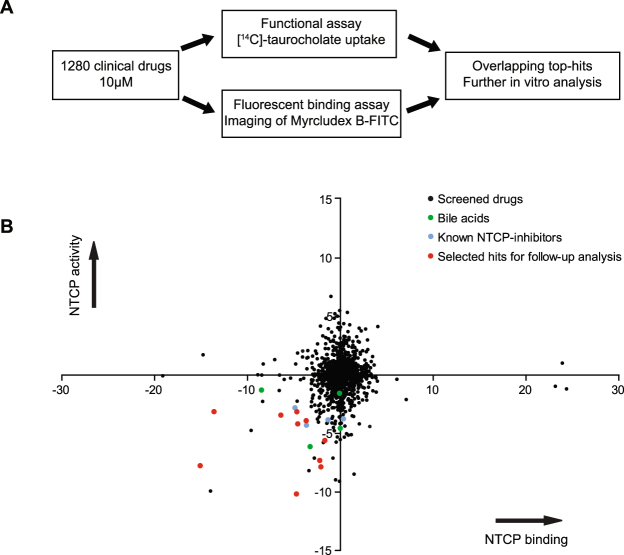
Screening approach and results. (**A**) Flow diagram of the approach to identify drugs that inhibit human NTCP. (**B**) B-scores for each compound of the two screening assays are depicted with inhibition of uptake present in the lower quadrants and inhibition of Myrcludex B binding in the left quadrants. Each dot represents a compound, with bile acids in green, previously known NTCP-inhibitors in blue, and the compounds selected for further analysis in red.

To find novel NTCP inhibitors, 1280 drugs were screened at a concentration of 10 µM (Fig. 1A). Therefore, two screening strategies using U2OS cells stably transfected with human NTCP were combined. Since U2OS cells do not express endogenous bile acid transporter proteins NTCP-independent taurocholate uptake activity is minimal^18,19^. Screening quality was assessed by defining the z-factor for each screening plate^20^, continuing data analysis for plates of a z-factor between 0.5–1.0. NTCP inhibition was acknowledged to a drug if taurocholate uptake or Myrcludex B-FITC fluorescence was decreased with at least 75% compared to the control. For each compound two B scores were calculated (Fig. 1B and Supplementary Table S1), which is a statistical score that shows the relative potency of a candidate within the studied population^21^. Amongst the top 150 hits, our screen identified bile acids (litocholic acid, chenodeoxycholic acid, ursodeoxycholic acid) (Fig. 1B, green dots) and drugs that had been described earlier as NTCP inhibitors (cyclosporin A, irbesartan, ezetimibe, pioglitazone^22–26^) (Fig. 1B, blue dots), which provided validation of both screening methods. From the top 30 we selected our hits, but we excluded bile acids and previously described NTCP inhibitors, as well as compounds inducing apoptosis or described as being hepatotoxic. We further narrowed down the remaining list of compounds by selecting one drug from a cluster of two or more drugs with a similar structure and/or mechanistic action, e.g. zafirlukast, pranlukast and montelukast. This resulted in a total of ten candidates selected from the lower-left quadrant for follow-up analysis: chicago sky blue 6B, flufenamic acid, nelfinavir mesylate hydrate, nifedipine, rosiglitazone, sulfasalazine, tolfenamic acid, toltrazuril, TRIAC, and zafirlukast (Fig. 1B red dots). Additionally, two compounds were selected that were only identified in one of the two screens: amlexanox (TC uptake screen) and hydroxytacrine maleate (Myrcludex B binding assay). Follow-up screening proved them to be false positives, demonstrating the effectiveness of using overlapping data from two different/orthogonal assays for compound selection.

### *In vitro* validation of selected overlapping top-hits

**Figure 2:**
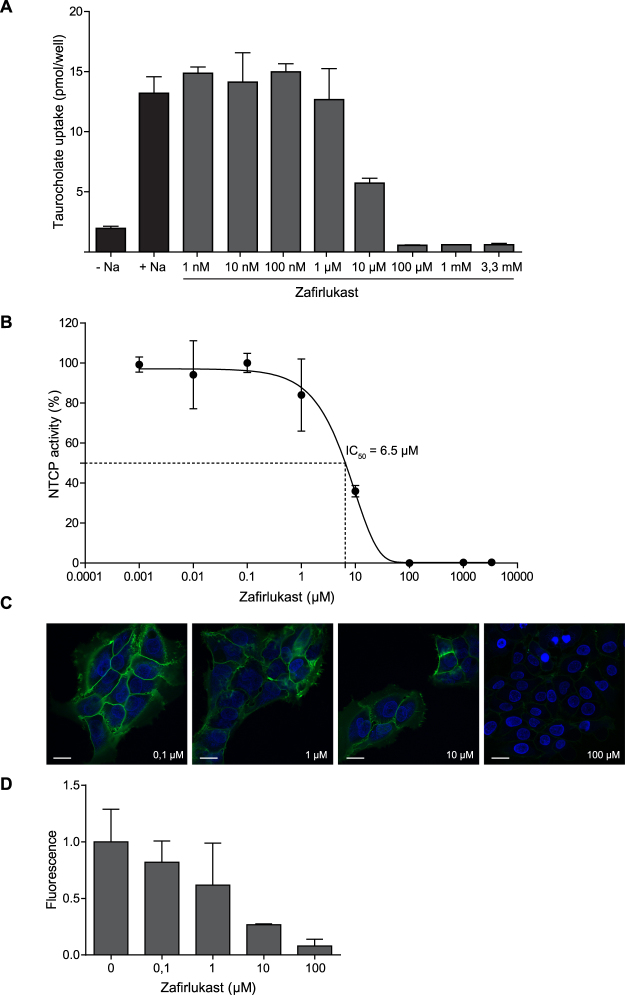
Inhibition of human NTCP by zafirlukast. (**A**) Taurocholate uptake into U2OS-HA-hNTCP cells was reduced in the presence of zafirlukast in a concentration-dependent fashion. (**B**) After normalization to maximal NTCP inhibition, the IC_50_ value was calculated. N = 2–4 wells/condition, experiment was repeated twice. (**C**) Representative confocal microscopy pictures of reduced Myrcludex B-FITC fluorescence upon co-administration with increasing amounts of zafirlukast. (**D**) Quantification of C, n = 3 per condition. All data are presented as mean ± SD.

**Figure 3:**
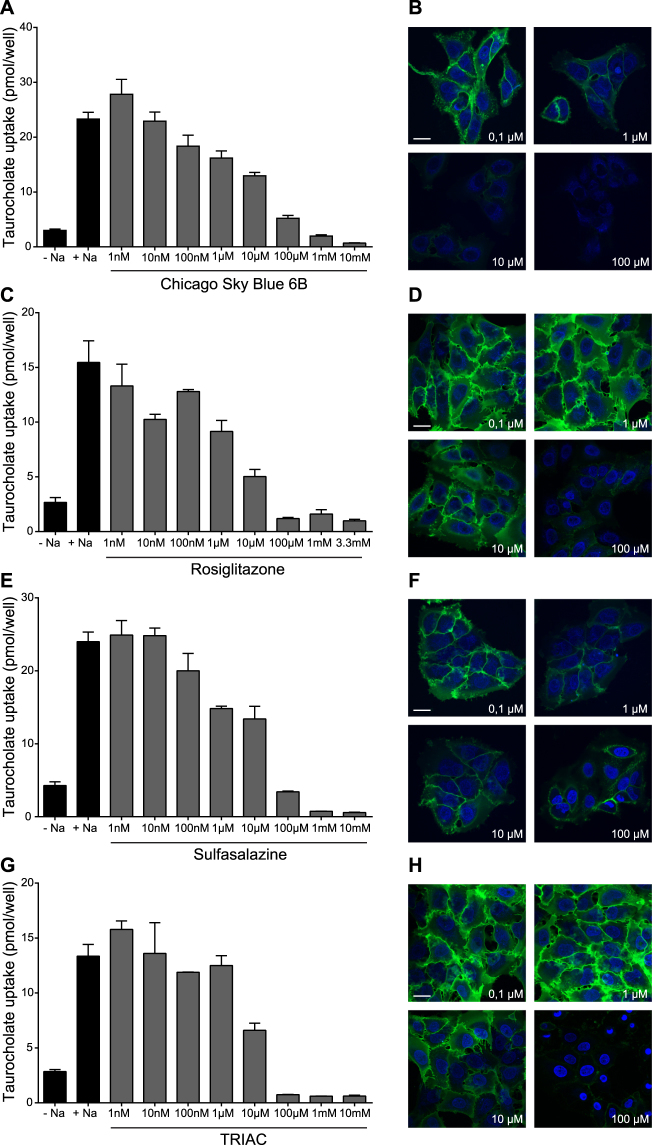
Inhibition of human NTCP by chicago sky blue 6B, rosiglitazone, sulfasalazine, and TRIAC. Taurocholate uptake and Myrcludex B binding was reduced to U2OS-HA-hNTCP cells in a concentration-dependent manner by chicago sky blue 6B (**A**,**B**), rosiglitazone (**C**,**D**), sulfasalazine (**E**,**F**), and TRIAC (**G**,**H**). Data are presented as mean ± SD, n = 2–4 wells/condition, experiment was repeated twice. From the confocal studies, representative pictures are shown, n = 3 per condition.

Five out of twelve selected compounds inhibited NTCP in repeated measurements of TC-uptake and Myrcludex B binding at concentrations of ≤ 10 µM: chicago sky blue 6B, rosiglitazone, sulfasalazine, TRIAC, and zafirlukast (Table 1). Inhibition was measured and IC_50_ values were calculated from increasing concentrations, ranging from 1 nM to 10 mM. Zafirlukast was one of the most potent inhibitors with an IC_50_ value of 6.5 µM (Fig. 2A and B) and more than 50% decreased Myrcludex B binding at 10 µM (Fig. 2C and D). Figure 3 shows the inhibitory effects of chicago sky blue 6B, rosiglitazone, sulfasalazine, and TRIAC on NTCP-mediated TC uptake and Mycludex B binding. Flufenamic acid and tolfenamic acid had an IC_50_ value of 10 and 12.5 µM respectively, and both decreased Myrcludex B binding to NTCP with 50% at ≤ 100 µM. Selected compounds with lower affinity were not included in further experiments (Supplementary Figures S1 and S2).

**Table 1:** Concentration-dependency of compounds to inhibit taurocholate uptake or Myrcludex B binding to human NTCP.

	IC_50_ for hNTCP-mediated TC uptake	≥50% loss of Myrcludex B binding to hNTCP
Rosiglitazone	5.1 µM	≥10 µM
Zafirlukast	6.5 µM	≥1 µM
TRIAC	6.9 µM	≥10 µM
Chicago Sky Blue 6B	7.1 µM	≥1 µM
Sulfasalazine	9.6 µM	≥1 µM
Flufenamic Acid	10 µM	≥100 µM
Tolfenamic Acid	12.5 µM	≥100 µM
Toltrazuril	84.1 µM	≥10 µM
Amlexanox	91.9 µM	≥100 µM
Nifedipine	99.6 µM	Not confirmed
Nelfinavir Mesylate Hydrate	134.2 µM	≥100 µM
Hydroxytacrine Maleate	4.2 mM	Not confirmed

Inhibition on taurocholate uptake was measured in U2OS-HA-hNTCP cells and IC_50_ values were calculated from increasing concentrations, ranging from 1 nM to 10 mM. Furthermore, with confocal microscopy for each compound it was determined at which concentration Myrcludex B binding was inhibited for minimally 50%. Compounds are arranged from low to high IC_50_ values.

### Myrcludex B inhibits uptake of tauro-ursodeoxycholic acid

**Figure 4:**
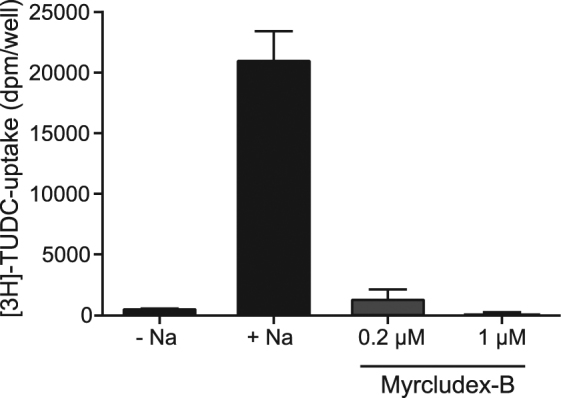
Myrcludex B inhibits uptake of tauro-ursodeoxycholic acid. Sodium dependent tauro-ursodeoxycholic acid uptake into U2OS-HA-hNTCP cells was reduced in the presence of Myrcludex B. N = 6 wells/condition, data are depicted as mean ± SD.

Ursodeoxycholic acid (UDCA), an unconjugated bile acid frequently used as treatment in cholestatic conditions, was found among the top 100 best hits (Supplementary Table S1). Indeed, sodium-dependent uptake of tauro-ursodeoxycholic acid is largely inhibited upon treatment with Myrcludex B (Fig. 4) as described before for taurocholic acid (IC_50_ 52,5 nM)^24^. This indicates that therapeutic co-administration of Myrcludex B at NTCP saturating conditions with UDCA might affect the pharmacokinetics of UDCA and vice versa. Of note, Myrcludex B effectively inhibits HBV/HDV infection at concentrations (83 pM for HBeAg in primary human hepatocytes^24^), where NTCP-mediated transport of substrates is not yet affected. From the other compounds that reduced NTCP activity (b-score ≤−5) frequent clinical co-application with Myrcludex B, and thus possibly drug-drug interaction, is not anticipated.

### Drug cytotoxicity

Drug cytotoxicity was examined for each compound at three concentrations. In U2OS-HA-hNTCP cells, viability was above 80% for all drugs at the two lowest concentrations (Supplementary Table S2). At 100 µM, nifedipine and zafirlukast exerted minor cytotoxicity, with a cell viability of 71.5 ± 7.3% and 77.7 ± 8.0% respectively. Similar cell viability results were obtained in U2OS-HA-hNTCP and in wildtype U2OS cells (Supplementary Table S2).

### Inhibition of HBV and HDV infection

**Figure 5:**
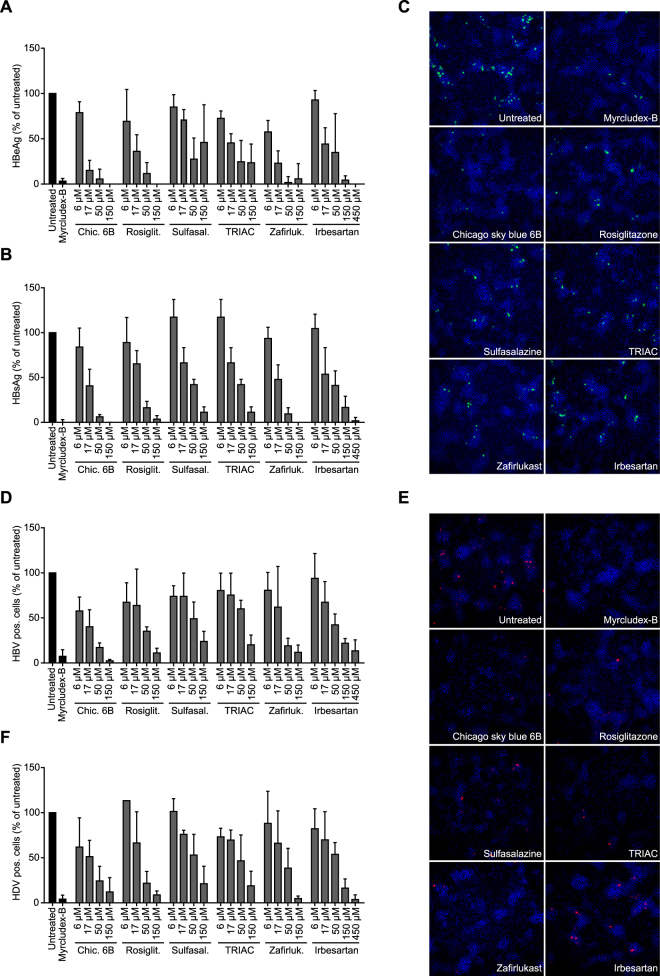
Inhibition of HBV and HDV infection in HepaRG cells. Rosiglitazone, sulfasalazine, TRIAC, zafirlukast, and irbesartan reduce both HBV (**A**–**D**) as well as HDV (**E**,**F**) infection in HepaRG cells. In a concentration-dependent fashion, hepatitis B extracellulair (**A**) and surface (**B**) antigen production was reduced by all compounds. Also, all compounds were effective in decreasing the amount of cells positively stained for HBcAg (**C**,**D**) and HDAg (**E**,**F**) in a concentration-dependent manner. Myrcludex B (1 µM) was included as positive control in all assays. Data are presented as mean ± SD, n = 3 × 2 wells/condition.

Since several NTCP inhibitors, e.g. irbesartan and cyclosporin A, have been shown to affect HBV and HDV infection^22–25,27^, we tested the 5 strongest hits from our screen in this experimental setting as well for interference with HBV/HDV infection. To that aim HepaRG cells were infected with HBV or HDV and viral markers HBsAg, HBeAg and intracellular HBcAg/HDAg were quantified. Myrcludex B and irbesartan were included as positive controls for infection inhibition. As depicted in Fig. 5A and B, production of both HBeAg and HBsAg was significantly reduced for all compounds in a concentration-dependent manner. Zafirlukast, chicago sky blue 6B and irbesartan were effective at 17 µM, whereas rosiglitazone, sulfasalazine, and TRIAC which were only effective in reducing HBsAg at concentrations of ≥50 µM. Higher concentrations of the drugs established a further reduction in HBeAg and HBsAg. Immunofluorescence data of hepatitis B core antigen (HBcAg) stained HepaRG cells at day 10 post HBV infection (Fig. 5C and D) corresponded to these findings: the number of positive stained cells was severely reduced by addition of 50 µM chicago sky blue 6B, rosiglitazone, or zafirlukast. At similar concentrations, sulfasalazine and TRIAC only induced a mild reduction in positive stained cells. For HDV infection, similar results were obtained (Fig. 5E and F). To address whether the inhibitory effect was limited to a specific HBV genotype or cell-derived virus we tested the effect of sulfasalazine using patient-derived HBV (genotype B), which was also from a different genotype than the cell derived HBV (genotype D). Sulfasalazine showed similar inhibitory effects on HBeAg and HBsAg levels for serum-derived HBV as for cell-derived HBV (Supplementary Figure S3), demonstrating that the inhibitory effects on infection were not limited to the source or genotype of HBV.

### Specificity for NTCP

**Figure 6:**
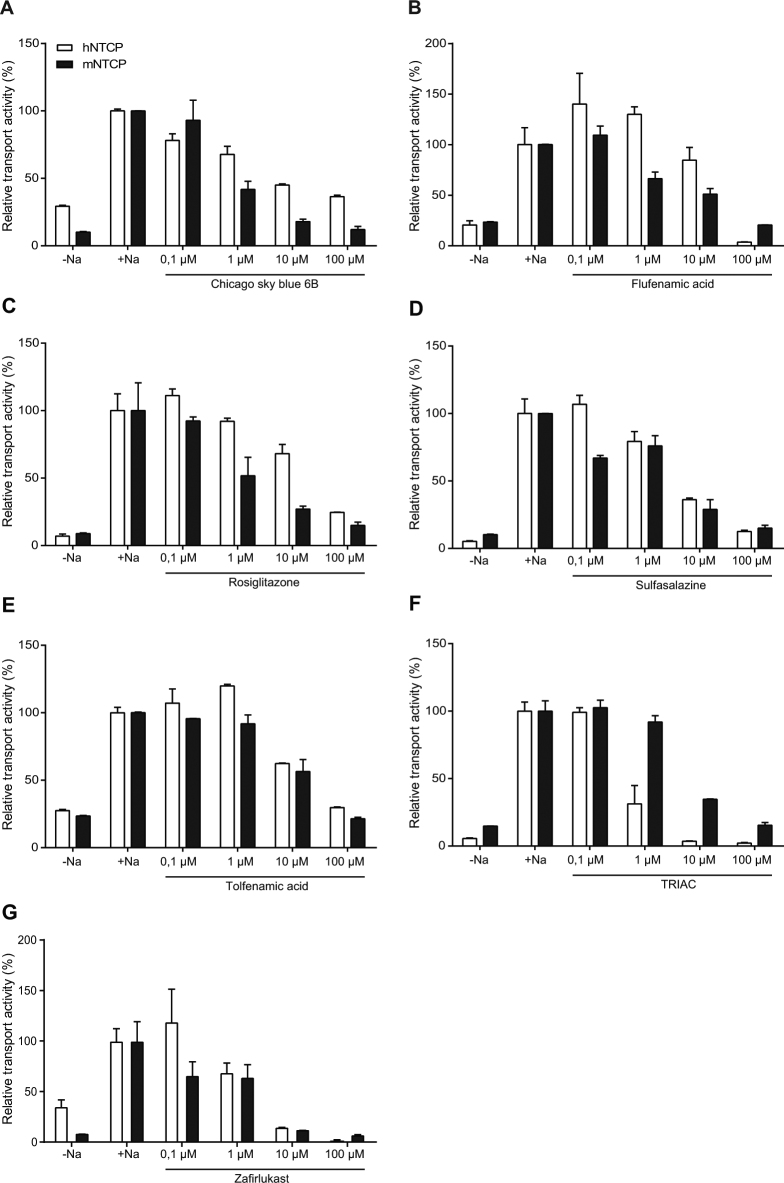
Compounds also inhibit mouse NTCP. (**A**–**G**) Taurocholate uptake into U2OS cells transiently transfected with mouse NTCP is inhibited similarly as human NTCP by sulfasalazine (**D**), tolfenamic acid (**E**) and zafirlukast (**G**), while chicago sky blue 6B (**A**), flufenamic acid (**B**), and rosiglitazone (**C**) are more potent in inhibiting mouse NTCP. TRIAC on the other hand inhibits human NTCP more strongly (**F**). Data are presented as mean ± SD, n = 2–4 wells/condition.

At the calculated IC_50_ value for hNTCP, chicago sky blue 6B, flufenamic acid, and rosiglitazone were more capable of inhibiting mouse Ntcp (Fig. 6A–C), while sulfasalazine, tolfenamic acid and zafirlukast were able to inhibit mouse Ntcp to a similar extent as human NTCP (Fig. 6D,E and G). TRIAC inhibited NTCP-mediated bile acid transport at lower concentrations for human NTCP than for mouse Ntcp (Fig. 6F).

**Figure 7:**
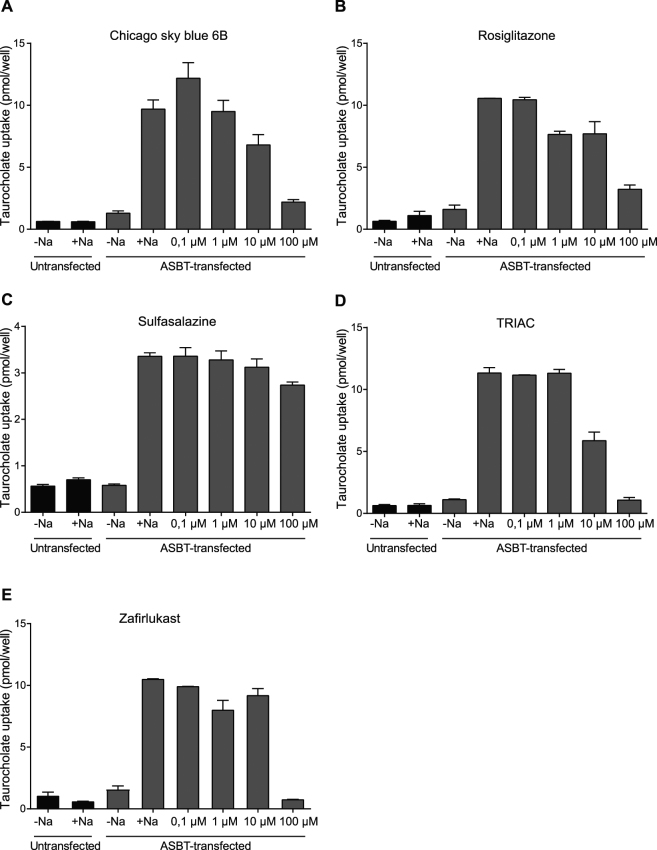
Some NTCP inhibitors also reduce ASBT-mediated bile acid uptake. (**A**–**E**) Sulfasalazine (**C**) is largely ineffective in inhibiting taurocholate uptake into MDCK-ASBT cells, while chicago sky blue 6B (**A**), rosiglitazone (**B**), TRIAC (**D**), and zafirlukast (**E**) inhibit ASBT in a concentration-dependent fashion. Data are presented as mean ± SD, n = 2–3 wells/condition.

The apical sodium bile acid transporter (ASBT, *SLC10a2*), most highly expressed at the brush border of ileal enterocytes^28^, is a closely related transporter showing highest sequence similarity with NTCP^1^. Both NTCP and ASBT mediate cellular uptake of conjugated bile acids. Therefore, we examined whether the 5 most potent NTCP inhibitors also affected ASBT-mediated bile acid transport. Both chicago sky blue 6B and TRIAC inhibited ASBT to a similar extent as NTCP with a comparable decrease in taurocholate uptake at all tested concentrations (Fig. 7A and D). Complete absence of ASBT inhibition was shown for sulfasalazine (Fig. 7C), while rosiglitazone and zafirlukast inhibited ASBT less effectively and at higher concentrations than observed for NTCP (Fig. 7B and E).

## Discussion

From 1280 clinically-applied drugs, this study identified 5 novel NTCP inhibitors that also block HBV and HDV infection *in vitro*. Until the recent identification of compounds that directly act on the viral large surface protein^29^, most HBV/HDV entry blockers identified to date inhibit both NTCP-mediated bile acid uptake as well as infection. Partially, this is explained by historical reasons as the inhibitory effect of compounds like cyclosporine^22,24^, ezetimibe^23^ and irbesartan^23,25^ on HBV infection was assessed when the receptor NTCP was identified and their effect on taurocholate uptake has already been known. However, the finding that also Myrcludex B, a peptide mimicking the myristoylated preS1-domain of the HBV envelope L-protein, interfered with NTCP-mediated bile acid uptake supported the notion that the HBV and bile acid binding sites in the NTCP protein are in close proximity and/or functionally intertwined^3^. In this study, the effects of 1280 clinically-applied compounds on both bile acid uptake and Myrcludex B binding were directly compared in an unbiased manner.

The screen confirmed NTCP inhibition by cyclosporin A, irbesartan, ezetimibe, and pioglitazone which had previously been identified as such^22–26^, and underscores the likelihood of a correlation between blocked bile acid transport and occupation of the HBV-binding site on NTCP. Reduced HBV/HDV infection *in vitro* was demonstrated for chicago sky blue 6B, rosiglitazone, sulfasalazine, TRIAC, and zafirlukast. In well-established HBV/HDV infection systems, these 5 novel inhibitors mainly act by targeting NTCP as the viral entry receptor. This is supported by the following two findings: 1) HDV infection was similarly affected as HBV infection. Both viruses share early infection events but follow different routes in viral replication^5,6^. 2) Cellular binding of Myrcludex B, the myristoylated preS1-domain of the HBV envelope L-protein^9^, was decreased upon co-administration with the 5 novel inhibitors, suggesting competition for the HBV binding site on the NTCP protein.

For these 5 compounds, bile acid transport is largely inhibited at a similar concentration needed to block HBV/HDV infection (IC_50_: 5–10 µM). On the contrary, Myrcludex B has an IC_50_ value for bile acid transport of 52.5 nM in primary human hepatocytes^24^, but blocks HBV infection at an at least 50-fold lower concentration (669 pM for HBsAg/83 pM for HBeAg), leaving a sufficient therapeutic range in which Myrcludex B efficiently blocks HBV/HDV infection while bile acid transport is largely unaffected. Another class of small molecules, proanthocyanidin and its analogues, were also shown to inhibit the HBV viral entry process with unaffected NTCP-mediated bile acid transport^29^. Proanthocyanidin directly targets the PreS1 region of the HBV L-protein, forming a novel class of anti-HBV agents.

The identification of 5 diverse novel NTCP inhibitors (chicago sky blue 6B, rosiglitazone, sulfasalazine, TRIAC, and zafirlukast) expands the chemical backbones to build new, more specific small molecule HBV/HDV entry inhibitors. Recently, cyclosporin A derivatives were developed that reduced NTCP-mediated HBV infection in primary hepatocytes by ~60% without significantly reducing bile acid uptake^30^. This suggests that analogues of rosiglitazone, sulfasalazine, zafirlukast and perhaps TRIAC could possibly be designed that more potently and specifically inhibit HBV infection. From the cheminformatics analysis limits for this chemical space can be established (Supplementary Figs S4 and S5). As ligands with a molecular weight <300 and AlogP of 3 or lower were identified, one could speculate that these ligands inhibit NTCP in a manner other than competitive inhibition of the bile acid binding site. Our virtual screening could not reliably identify novel active compounds. Unlike other bile acid transporters like OATPs, NTCP is likely not addressed by most chemotypes, as the percentage of actives identified in this study is much lower than in a similar screen for OATP inhibitors (respectively ~1% versus 7–10%)^31^. Previous computational models further support this observation^23,25,26,32,33^. Although they all could elucidate the requirement of hydrophobes and hydrogen bond acceptors, the exact number for these features varied between 3 and 1 for both in line with the current work^23,26,32,33^. Furthermore, Kramer *et al*.^34^ concluded that substrate specificity is much broader for NTCP than for the related bile acid transporter ASBT. Indeed, sulfasalazine, rosiglitazone, and zafirlukast, are more selective for NTCP, while chicago sky blue 6B and TRIAC were as effective for ASBT as for NTCP. Chicago sky blue 6B was also identified as an inhibitor for vesicular glutamate transporters (VGLUTs), completely unrelated transporters, further demonstrating its low specificity^35^. Although species-specific differences have been found for drugs bosentan^36^ and rosuvastatin^37^, only minor differences in drug specificity between mouse and human NTCP were found for sulfasalazine, rosiglitazone, and zafirlukast.

The usage of clinically-relevant compounds in a screen for NTCP inhibitors provides additional information as it seems likely that several of the hits are NTCP substrates. Myrcludex B could possibly be used in combination with such drugs, potentially leading to drug-drug interactions. In this respect, predominantly ursodeoxycholic acid (UDCA) should be considered. UDCA is an unconjugated bile acid frequently used as treatment in cholestatic conditions, mainly primary biliary cholangitis^38^. Upon administration, all UDCA is converted to its glycine and taurine conjugated forms which mediate its hepatoprotective effects^39^. This therapeutic bile acid was found amongst the top 100 hits and Myrcludex B largely inhibited NTCP-dependent uptake of tauro-UDCA. This suggests that Myrcludex B co-administration might affect clearance and exposure of (conjugated) UDCA at dosages that interfere with the transporter function of NTCP, which are higher than the dose required for lowering HBV/HDV infection.

Supplementary Table S4 describes expected serum levels of sulfasalazine, rosiglitazone, TRIAC, and zafirlukast upon normal therapeutic use for their intended diseases^40–44^. With their IC_50_ for NTCP in mind, sulfasalazine, rosiglitazone, and TRIAC are relevant to be considered as NTCP inhibitors upon normal therapeutic use, while treatment with zafirlukast is unlikely to induce NTCP inhibition. Notably, sulfasalazine has previously been shown to be a potent inhibitor of glycochenodeoxycholic acid (GCDCA)- induced apoptosis in hepatocytes *in vitro* and in the intact liver independently of its inhibiting effects on hepatocellular GCDCA uptake^50^, making it even more attractive as a candidate for therapeutic NTCP inhibition. In conclusion, from a library of clinically-applied drugs various compounds were identified that inhibit NTCP-mediated bile acid uptake and HBV/HDV infection *in vitro*. These findings could contribute to the development of novel anti-HBV and HDV agents.

## Materials and Methods

### Chemicals

The Prestwick Chemical Library® containing 1280 approved drugs as 10 mM stock solutions in 96-well plates was purchased from Prestwick (Prestwick Chemical, Illkirch, France). Amlexanox was purchased from Abcam. Hydroxytacrine Maleate was obtained from Santa Cruz Biotechnology (Santa Cruz, USA). Chicago Sky Blue 6B, Flufenamic acid, Nelfinavir Mesylate Hydrate, Nifedipine, Rosiglitazone, Sulfasalazine, Tolfenamic acid, Toltrazuril, 3,3′,5-Triiodothyroacetic acid (TRIAC), Zafirlukast, and Taurocholic acid (TC) were bought from Sigma-Aldrich (Zwijndrecht, The Netherlands). Myrcludex B and Myrcludex B-FITC were customly synthesized by Pepscan (Lelystad, The Netherlands). Hoechst 33342 was obtained from Merck Millipore (Darmstadt, Germany). [^3^H]Taurocholic acid (1 mCi/ml) and [^14^C]Taurocholic acid (0.05 mCi/ml) were purchased from PerkinElmer (Groningen, The Netherlands).

### Cell culture

U2OS and MDCK cells were grown in Dulbecco’s modified Eagle’s medium (DMEM, Sigma-Aldrich), supplemented with 10% FCS (Gibco), 1% L-glutamine (Lonza), and 1% penicillin/streptomycin (Lonza). Medium of U2OS cells stably transfected with human NTCP (U2OS-HA-hNTCP)^18^ or MDCK cells stably expressing human ASBT (MDCK-hASBT; kind gift of Paul Dawson)^45^ was supplemented with 400 µg/ml or 350 µg/ml Geneticin (Invitrogen) respectively. Transient transfection of U2OS cells with mouse NTCP (mNTCP) was done with PEI reagent (Brunschwig, Basel, Switzerland). Shortly, U2OS cells were seeded one day prior to transfection in 24-well plates. On the day of transfection, 0.5 µg DNA was mixed with 100 µl unsupplemented DMEM and 3 µg/µl PEI. After 10–15 minute incubation at RT, 20 µl of the transfection mix was added to each 24-well. Transfected cells were used in further experiments 48–72 hours later. Cell lines were passaged twice a week at a confluence of 80%, and incubated in a humidified atmosphere of 37 °C + 5% CO_2_.

HepaRG cells were cultured in Williams E medium supplemented with 10% heat-inactivated fetal calf serum, 2 mM L-glutamine, 5 µg/ml insulin, 50 µM hydrocortisone, 50 U/ml penicillin, and 50 µg/ml streptomycin. After two weeks of cultivation, differentiation was induced by 1.5% dimethyl sulfoxide (DMSO) as described^11^.

### Screening studies

We screened the Prestwick Chemical Library® in 96-well format using two different screening assays: a taurocholate uptake assay (using PerkinElmer ViewPlate-96 white microplate clear bottom, product number 6005181) and a competitive binding assay (using PerkinElmer CellCarrier-96 black, product number 6005550). Plates were seeded with U2OS-HA-hNTCP (92 wells) or U2OS (4 wells, background) cells at a density of 50.000 cells/well or 10.000 cells/well for the functional or the competitive binding assay respectively. Screening assays were performed at room temperature approximately 24 hours later. Drug concentration was 10 µM and 1 µM Myrcludex B was used as positive control for NTCP inhibition and binding.

For the taurocholate uptake studies, medium was aspirated and cells were washed once with 100 µL uptake buffer: 5 mM KCl, 1.1 mM K_2_HPO4, 1 mM MgCl_2_, 1.8 mM CaCl_2_, 10 mM D-Glucose, 10 mM HEPES and 136 mM NaCl. For sodium free uptake buffer, NaCl was replaced by 136 mM NMDG. Buffers were set to pH 7.4, at room temperature. After washing, cells were pre-incubated for 10 minutes with drugs in 50 µL uptake buffer. Subsequently, 50 µL uptake buffer containing [^14^C]taurocholate was added to the wells for another 10 minutes. After incubation, buffer was removed and cells were washed 2x with ice-cold PBS. Cells were lysed for a minimum of 30 minutes in 30 µL/well 0.05% SDS in water. When lysis was complete, 120 µL MicroScint™-20 (PerkinElmer) was added to the wells and radioactivity was measured by liquid scintillation counting.

For the fluorescent binding assay, medium was aspirated and cells were washed once with 100 µL PBS. A 30 minute incubation period followed in which all cells were incubated with 0.2 µM Myrcludex B-FITC with or without drug in 100 µL Leibovitz’s L-15 Medium (Invitrogen). Cells were washed twice with PBS and subsequently 100 µL Leibovitz’s L-15 Medium was added. Of each well, 9 photos (10x objective) were taken in the brightfield and FITC channel using the Operetta CLS™ High-Content Imaging System (PerkinElmer).

### Inhibition studies

Wildtype U2OS or U2OS-HA-hNTCP were seeded at a density of 50.000 cells in 500 µL complete medium in 24-well plates (VWR® Cat. Number 734–2325). For MDCK or MDCK-hASBT cells 70.000 cells/well were plated in 24-well plates, followed by 24 hours 10 mM sodium butyrate (Sigma-Aldrich) stimulation the next day. Cells were used in the inhibition studies 24 hours (U2OS cell lines) or 48 hours (MDCK cell lines) later. Inhibition studies with mNTCP transiently transfected cells were conducted 48–72 hours after transfection.

Medium was aspirated from the wells and cells were washed once with 250 µL uptake buffer. Subsequently, cells were pre-incubated with drug in 150 µL uptake buffer for 10 minutes at 37 °C. Taurocholate uptake was initiated by addition of 100 µL uptake buffer containing taurocholate (final concentration 10 µM TC spiked with tritium-labeled TC) and cells were incubated for 2 minutes at 37 °C. Subsequently, cells were washed 4 times with ice-cold PBS and lysed with 0.05% SDS in water. Radioactivity was measured by liquid scintillation counting.

### Confocal microscopy

Wildtype U2OS or U2OS-HA-hNTCP were seeded in a sterile 8-well coverslip bottomed chamber slide (Thermo Fisher Scientific, 155411). After 24 hours, medium was aspirated and cells were washed once with 300 µl Leibovitz’s L-15 Medium (Invitrogen). A 30 minute incubation period followed in which all cells were incubated with 0.2 µM Myrcludex B-FITC with or without drug (1, 10, 100 µM) and Hoechst (1/1000) in 300 µL Leibovitz’s L-15 Medium. Cells were washed twice with Leibovitz’s L-15 Medium and subsequently 400 µL Leibovitz’s L-15 Medium was added. Live cell fluorescent imaging was performed using a Leica SP8X-SMD confocal microscope with fully enclosed incubation at 37 °C. A 63x oil objective was used and images were captured while the cells were consecutively excited at 490 nm (FITC) 350 nm (Hoechst). ImageJ software (National Insititutes of Health, Bethesda, MD) was employed for data analysis.

### Cytotoxicity studies

Drug cytotoxicity was evaluated by measuring the cell viability using the water soluble tetrazolium salt WST-1 (Sigma-Aldrich). Shortly, U2OS-HA-hNTCP cells were seeded at a density of 10.000 cells/well in 96-well plates (Corning® Costar® Cat. Number 3595), and incubated for approximately 24 hours at 37 °C + 5% CO_2_. Medium was aspirated and cells were exposed to 1 µM, 10 µM, or 100 µM drug in 100 µL complete medium. Empty wells containing only complete medium served as background. In addition, each well was supplemented with 10 µL WST-1. After two hours incubation (37 °C + 5% CO_2_), absorbance at 450 nm and 690 nm was measured using a Synergy HT Multi-Mode Microplate Reader (Biotek). Cell viability was calculated using the following formula: (A_450_-A_690_ (sample)) − (A_450_-A_690_ (background)). Viability levels higher than 80% were considered healthy and unaffected by the drug.

### HBV/HDV infection studies

HBV was produced in HepAD38 cells^46^. HDV was produced by co-transfection of HuH7 cells with the two plasmids pSVLD3 (HDV genotype 1, kindly provided by John Taylor^47^) and pT7HB2.7 (HBV genotype D, kindly provided by Camille Sureau^48^). Virions in the culture supernatant were purified and concentrated by heparin affinity chromatography on an ÄKTApurifier FPLC system (GE Healthcare) and stored at −80 °C in 10% FCS.

Cells were pre-incubated for 30 min with NTCP inhibitors or Myrcludex B in medium containing 4% polyethylene glycol (PEG) 8000 (Sigma-Aldrich) and 1.5% DMSO. They were then inoculated with HBV or HDV for 14 h at 37 °C. After inoculation, cells were washed twice with PBS and post-incubated with NTCP inhibitors or Myrcludex B. After 10 hours, the cells were washed twice in PBS before medium supplemented with 1.5% DMSO was added. Medium was exchanged every 2 days. Cells infected with HDV were fixed on day 5 post infection and HDV infection was quantified by immunofluorescence. To quantify the HBV infection, secretion of hepatitis B surface antigen (HBsAg) and hepatitis B extracellular antigen (HBeAg) into the culture supernatant from day 7 to 10 post infection was quantified by enzyme-linked immunosorbent assay (ELISA). The cells were fixed 10 days post infection and analysed by hepatitis B core antigen (HBcAg) specific immunofluorescence. For HBsAg-ELISA, 96 well ELISA microplates (Greiner bio-one) were coated with monoclonal mouse anti-HBsAg (Fitzgerald) and were then blocked with 2% BSA and 0.05% Tween-20 in PBS. After blocking, supernatant from infected cells was added (1:10 dilution in PBS). Plates were incubated with biotinylated monoclonal mouse anti-HBsAg (Fitzgerald). The biotinylation was performed using EZ-Link Sulfo-NHS-LC-Biotin (ThermoFisher) as suggested by the provider. Pierce high sensitivity Streptavidin-HRP (ThermoFisher) was added for 30 min at 37 °C. The plates were washed three times with wash buffer (0.05% Tween-20 in PBS) after incubation with supernatant samples, biotinylated antibodies and Streptavidin-HRP. For HBeAg-ELISA, 96 well ELISA microplates were coated with monoclonal mouse anti-HBeAg (Fitzgerald) and blocked in PBS with 2% BSA and 0.05% Tween-20. After blocking, supernatant samples were addedMouse monoclonal anti-HBeAg-HRP (Fitzgerald) was added. The plates were washed three times with wash buffer after incubation with supernatant samples and HRP-conjugated antibodies. A dilution series of supernatant from HepAD38 cells was used as standard curve. The reaction was visualized by addition of TMB substrate (eBioscience) and stopped with 1 M H_3_PO_4_. Absorbance at 450 nm was measured with a PerkinElmer EnVision multilabel reader. For immunofluorescence HDV- and HBV-infected HepaRG cells were fixed with 4% paraformaldehyde in PBS 5 or 10 days post infection, respectively. Cells were permeabilized with 0.25% (vol/vol) Triton X-100 in PBS. VUDA serum anti-HDAg or rabbit-anti-HBcAg (DAKO) were used as primary antibodies. For quantification of infection, 4 images per treatment were acquired with an epifluorescence microscope and HDAg- or HBcAg-positive cells were quantified with ilastik software^49^.

### Statistics

Screen data were analysed by R software^21^ for statistical computing. Variance was adjusted by plate and data were normalized to positive and negative controls, resulting in a B-score for each sample. Follow-up data are provided as the mean ± standard error of the mean. Graphpad Prism 5 was used for statistical analysis. Significance levels were calculated using the Student’s t-test and results were considered significant at p < 0.05. IC_50_ values were calculated with non-linear regression curves.

## Electronic supplementary material

Supplementary data

Supplementary Table 1
