# Supplementary material for: Reduced hepatitis B and D viral entry using clinically applied drugs as novel inhibitors of the bile acid transporter NTCP
Source: Sci Rep. 2017 Nov 10;7:15307. doi: 10.1038/s41598-017-15338-0 (PMC5681660; doi:10.1038/s41598-017-15338-0)
Supplement: Supplementary file 1 — Supplementary data [file 41598_2017_15338_MOESM1_ESM.doc]

**Reduced hepatitis B and D viral entry using clinically applied drugs as novel inhibitors of the bile acid transporter NTCP**

Joanne M. Donkers^1^, Benno Zehnder^2^, Gerard J.P van Westen^3^, Mark J. Kwakkenbos^4^, Adriaan P. IJzerman^3^, Ronald P.J. Oude Elferink^1,5^, Ulrich Beuers^1,5^, Stephan Urban^2,6^, Stan F.J. van de Graaf^1,5^

^1^Tytgat Institute for Liver and Intestinal Research & ^5^Department of Gastroenterology & Hepatology, Amsterdam Gastroenterology and Metabolism, AMC, Amsterdam, The Netherlands

^2^Department of Infectious Diseases, Molecular Virology, University Hospital Heidelberg, Heidelberg, Germany

^3^Medicinal Chemistry, Leiden Academic Centre for Drug Research, Leiden University, Leiden, The Netherlands
^4^Aimm Therapeutics, Amsterdam, The Netherlands

^6^German Center for Infection Research, Heidelberg University, Heidelberg, Germany

**Supplementary materials and methods***Serum-derived HBV genotype B infection study*For these experiments HepaRG cells stably transduced with hNTCP were used. Differentiation and serum-derived HBV genotype B infection were performed following similar protocols as described for wildtype HepaRG cells in the main materials&methods with the following changes: 8 hours infection and supernatant collection for HBeAg and HBsAg analysis from day 9 to 12.
Total RNA was extracted from cells 12 days after infection using the NucleoSpin RNA isolation kit (Macherey-Nagel). Reverse transcription was performed with the High-Capacity cDNA Reverse Transcription Kit (Thermo Scientific). mRNA levels were determined by qPCR using the iTaq™ Universal SYBR® Green Supermix (Bio-rad) with HBV specific primers (forward: 5’-GTTGCCCGTTTGTCCTCTAATTC-3’; reverse: 5’-GGAGGGATACATAGAGGTTCCTTGA-3’). GAPDH mRNA levels were used for normalization (forward: 5’-ACCCAGAAGACTGTGGATGG-3’; reverse: 5’-TCTAGACGGCAGGTCAGGTC-3’).
Total DNA was extracted from cells 12 days after infection using the NucleoSpin Tissue kit (Macherey-Nagel). HBV DNA levels were determined by qPCR PerfeCTa qPCR Toughmix (Quanta Biosciences) with HBV specific primers (forward: 5’- GTGTCTGCGGCGTTTTATCA-3’; reverse: 5’- GACAAACGGGCAACATACCTT-3’) and a probe (5’-TxRed-AGGGTTGGCCAATCTACTCC-BHQ2-3’). β-globin levels were used for normalization (forward: 5’- GAAGAGCCAAGGACAGGTAC-3’; reverse: 5’- TGAGGTTGCTAGTGAACACAG-3’; probe: 5’-HEX- AGGGTTGGCCAATCTACTCC-BHQ1-3’).

*Cheminformatics analysis*

Work was performed in Pipeline Pilot version 2016. Actives were the 10 strongest hits (average value from binding assay and functional assay < 25). Hits from the current work were combined with NTCP ligands from literature trough the ChEMBL database (version 20). For public domain ligands actives were defined as having a pChembl value (pIC50, pKi, pEC50) of better than 5 (10 μM) or 25% activity remaining. In total this led to a set of 1312 ligands, 32 actives and 1280 inactives. After pre-processing (standardization, stereochemistry standardization, hydrogen addition), molecules were described by physicochemical properties calculated in Pipeline Pilot (AlogP, molecular weight, number of hydrogen bond donors and acceptors, molecular polar surface area, number of aromatic rings, and molecular fractional polar surface area) and circular fingerprints (functional class extended connectivity with a diameter of 4 bonds, FCFP_4)^2^. The principal component analysis was and the quantitative structure-activity relationship (QSAR) model were performed in R statistics. For the QSAR model Random Forest based machine learning was used^3^. The performance of five QSAR models, trained with a different split of training set (for model fit) and test set (for model validation), was averaged to obtain a performance estimate. Physicochemical properties were binned for improved interpretability (Supplementary Table S3). Primary performance metric was the Matthews Correlation Coefficient (MCC)^4^.

**Supplementary results**

*Serum-derived HBV genotype B infection study* In order to investigate if the source and genotype of HBV influenced the inhibitory effects of one of the 5 novel inhibitors, HepaRG cells overexpressing NTCP were infected with patient-derived HBV, genotype B, and treated with sulfasalazine. Viral markers HBeAg and HBsAg were quantified by chemo-luminescence and ELISA respectively. HBV DNA and HBV RNA were measured by quantitative PCR. Myrcludex B was included as positive control for infection inhibition. Sulfasalazine reduced HBeAg, HBsAg, HBV DNA, and HBV RNA in a dose-dependent fashion (Supplementary Fig. S3).

*Cheminformatics analysis*

A principal component analysis was performed indicating that NTCP inhibitors tend to have a molecular weight over 350 and an AlogP of over 3. However solely physicochemical properties cannot explain drug binding as actives are not clustering tightly together but are located within groups of drugs not binding to NTCP (Supplementary Fig. S4A). The same was true for other compounds and a principal component analysis performed on the data (Supplementary Fig. S4B). However, box and whisker plots for several properties demonstrated distinct differences between actives and inactives (Supplementary Fig. S5A-G). Hence, a QSAR model was trained on physicochemical properties and chemical substructures as was done previously in order to further understand properties guiding NTCP binding^1^.

QSAR models were predictive, in external validation they obtained an average MCC of 0.50 (±0.11), sensitivity of 0.73 (±0.21), specificity 0.97 (±0.01), Receiver-Operator Characteristic of 0.93 (±0.07), and an overall accuracy of 0.97 (±0.01) (Supplementary Fig. S4C). Hence models were interpreted to understand drug properties guiding NTCP inhibition. Drugs inhibiting NTCP are found to be similar to bile acids. Chemical substructures identified to be positively correlated to inhibition in the QSAR model include parts of bile acid chemical scaffolds. Moreover, the QSAR confirms that NTCP inhibitors tend to be large lipophilic compounds but more importantly identifies a number of chemical substructures that important for ligand binding (Supplementary Fig. S4D). The obtained model was used to virtually screen a chemical vendor database, eMolecules, but no other potential ligands were obtained than those very highly similar to ligands in the training set. This could be caused by a lack of potential ligands in the eMolecules database or by a failure of the QSAR to retrieve actives.

**Supplementary Table S1: Results of the initial screening**

Data is included in the Supplementary Excel file.

For each compound the results of both screening assays are shown in percentages compared to the untreated control and as statistical B-score. For the B-score calculation, variance was adjusted by plate and results were normalized to positive and negative controls. Per screen compounds were ranked 1-1280 with 1 as strongest inhibitor. Scores were combined by multiplying the root of both ranks. Compounds are listed in order of lowest combined ranking score.

|  | U2OS-HA-hNTCP cells | | | U2OS cells | | |
| --- | --- | --- | --- | --- | --- | --- |
|  | **1 µM** | **10 µM** | **100 µM** | **1 µM** | **10 µM** | **100 µM** |
| **Amlexanox** | 99.6 ± 6.1 | 101.8 ± 18.9 | 92.7 ± 17.8 | 88.2 ± 9.6 | 94.8 ± 13.8 | 77.3 ± 5.6 |
| **Chicago Sky Blue 6B** | 87.8 ± 7.2 | 95.8 ± 5.9 | 110.9 ± 9.4 | 91.0 ± 9.3 | 93.9 ± 5.4 | 108.7 ± 5.6 |
| **Flufenamic Acid** | 125.1 ± 15.2 | 117.9 ± 9.6 | 106.9 ± 9.6 | 108.7 ± 9.1 | 107.5 ± 8.5 | 104.3 ± 3.6 |
| **Hydroxytacrine Maleate** | 105.6 ± 1.2 | 102 ± 8.3 | 96.7 ± 5.9 | 107.6 ± 15.7 | 100.7 ± 7.1 | 95.8 ± 3.9 |
| **Nelfinavir Mesylate Hydrate** | 105.3 ± 7.1 | 99.8 ± 5.2 | 104.8 ± 4.5 | 105.9 ± 8.4 | 102 ± 10.7 | 95.1 ± 5.6 |
| **Nifedipine** | 90.1 ± 4.9 | 89.2 ± 9.6 | 71.5 ± 7.3 | 100.6 ± 7.4 | 107.9 ± 7.1 | 88.4 ± 11.7 |
| **Rosiglitazone** | 111.0 ± 4.4 | 106.0 ± 4.5 | 103.1 ± 6.0 | 104.2 ± 7.2 | 107.0 ± 3.4 | 103.9 ± 2.5 |
| **Sulfasalazine** | 98.1 ± 3.0 | 90.6 ± 6.9 | 85.0 ± 9.1 | 100.5 ± 6.9 | 100.9 ± 8.4 | 97.8 ± 8.0 |
| **Tolfenamic Acid** | 116.1 ± 10.3 | 117.8 ± 13.0 | 106.8 ± 12.4 | 94.7 ± 6.3 | 104.9 ± 11.7 | 82.1 ± 4.8 |
| **Toltrazuril** | 100.4 ± 9.9 | 109.4 ± 12.9 | 105.4 ± 11.4 | 114.8 ± 6.1 | 109.3 ± 11.8 | 100.9 ± 3.6 |
| **TRIAC** | 108.6 ± 4.1 | 104.9 ± 2.1 | 86.3 ± 4.9 | 100 ± 7.5 | 99.8 ± 5 .0 | 81.6 ± 3.8 |
| **Zafirlukast** | 94.7 ± 12.7 | 87.1 ± 12.3 | 77.7 ± 8.0 | 87.7 ± 4.8 | 94.1 ± 2.7 | 73.0 ± 5.8 |
| **Supplementary Table S2: Drug cytotoxicity studies**  U2OS-HA-hNTCP and U2OS cell viability by WST-1 assay was evaluated 2 hours after drug administration. Each drug was tested at three concentrations. Data is expressed as mean ± SD, n= 3. | | | | | | |

| **Property** | **Low** | **Medium** | **High** |
| --- | --- | --- | --- |
| Molecular Polar Surface Area | < 76 | 76-150.99 | > 150.99 |
| AlogP | <0.01 | 0.01 - 5 | >5 |
| Molecular Weight | <300 | 300-500 | >500 |
| Num H Donors | <3 | 3-5 | >5 |
| Num H Acceptors | <5 | 5-10 | >10 |
| Num Aromatic Rings | <1 | 1-2 | >2 |
| Num Rotatable Bonds | <3 | 3-9 | >9 |
| **Supplementary Table S3: Cut-offs used to bin physicochemical properties**  Physicochemical properties were binned for improved interpretability of the cheminformatics analysis. | | | |

| **Drug** | **Therapeutic dose** | **Expected serum concentration** | **NTCP IC_50_** |
| --- | --- | --- | --- |
| Sulfasalazine | 2-4 gram/day | 1 gram 🡪 6 µg/ml (~15 µM)^5^ | 9.6µM |
| Rosiglitazone | 4-8 mg/day | 8 mg 🡪 600 ng/ml (~1.7 µM)^6^ | 5.1µM |
| TRIAC | 500-1000 mg/day | 1000 mg 🡪 ~215 µM (with absorption 67%)^7^ | 6.9µM |
| Zafirlukast | 40 mg/day | 20 mg 🡪 326 ng/ml (~0.6 µM)^8^ | 6.5µM |
| **Supplementary Table S4:** **Therapeutic dose, expected serum concentration in comparison to the IC_50_ of NTCP for sulfasalazine, rosiglitazone, TRIAC and zafirlukast.** For each drug the therapeutic dose, expected serum concentration and the IC_50_ of NTCP are displayed. A normal therapeutic dose of sulfasalazine, rosiglitazone and TRIAC can potentially inhibit NTCP, while treatment with zafirlukast is unlikely to induce NTCP inhibition. | | | |


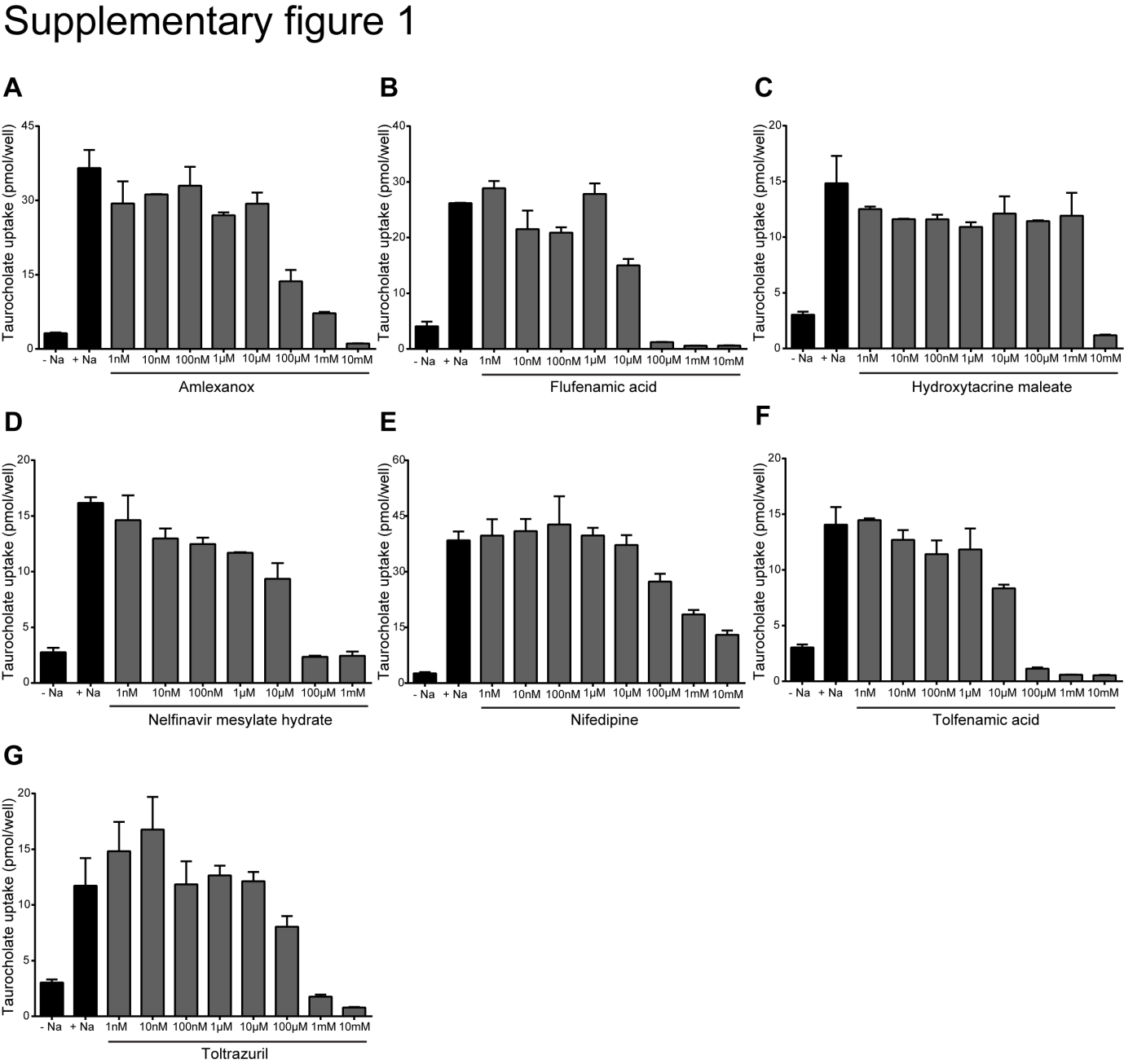


**Supplementary Figure S1. Secondary screening results of novel NTCP inhibitors.** (A-G) Taurocholate uptake into U2OS-HA-hNTCP cells was reduced in a dose-dependent fashion by amlexanox (A), flufenamic acid (B), nelfinavir mesylate hydrate (D), nifedipine (E), tolfenamic acid (F), and toltrazuril (G), but not by hydroxytacrine maleate (C). Cells were incubated with taurocholate with tracer amounts of [^3^H]TC for 2 minutes, 37˚C, and tritium activity (dpm) was measured in cell lysates. All data are presented as mean ± SD, n=2-4 wells/condition, experiments were repeated twice.

**
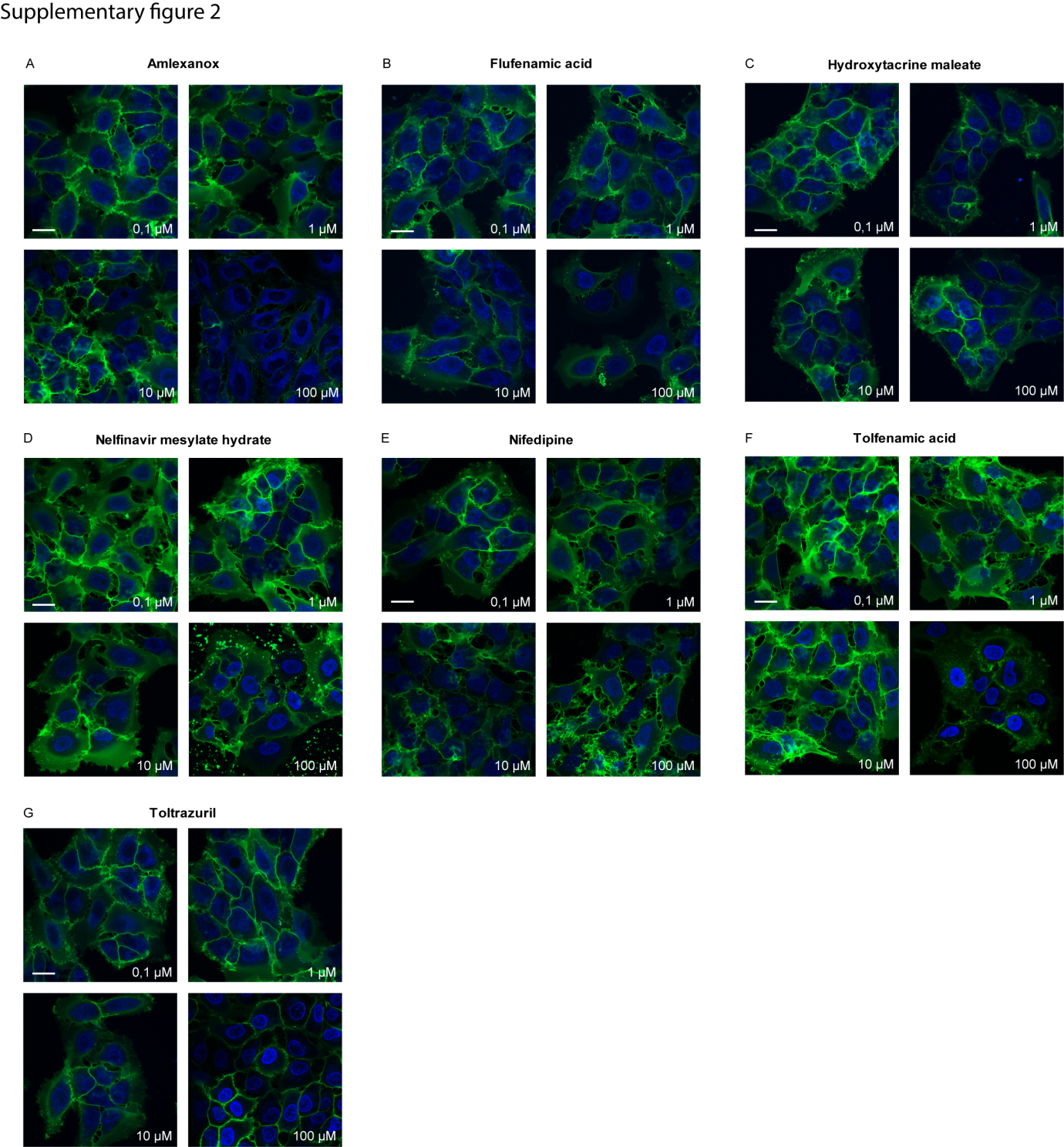
**

**Supplementary Figure S2. Confocal microscopy with novel NTCP inhibitors.** (A-G) Representative confocal microscopy pictures of reduced Myrcludex B-FITC fluorescence upon co-administration with increasing amounts of amlexanox (A), flufenamic acid (B), nelfinavir mesylate hydrate (D), tolfenamic acid (F), and toltrazuril (G), but not by hydroxytacrine maleate (C) and nifedipine (E). N=3 per condition.

**
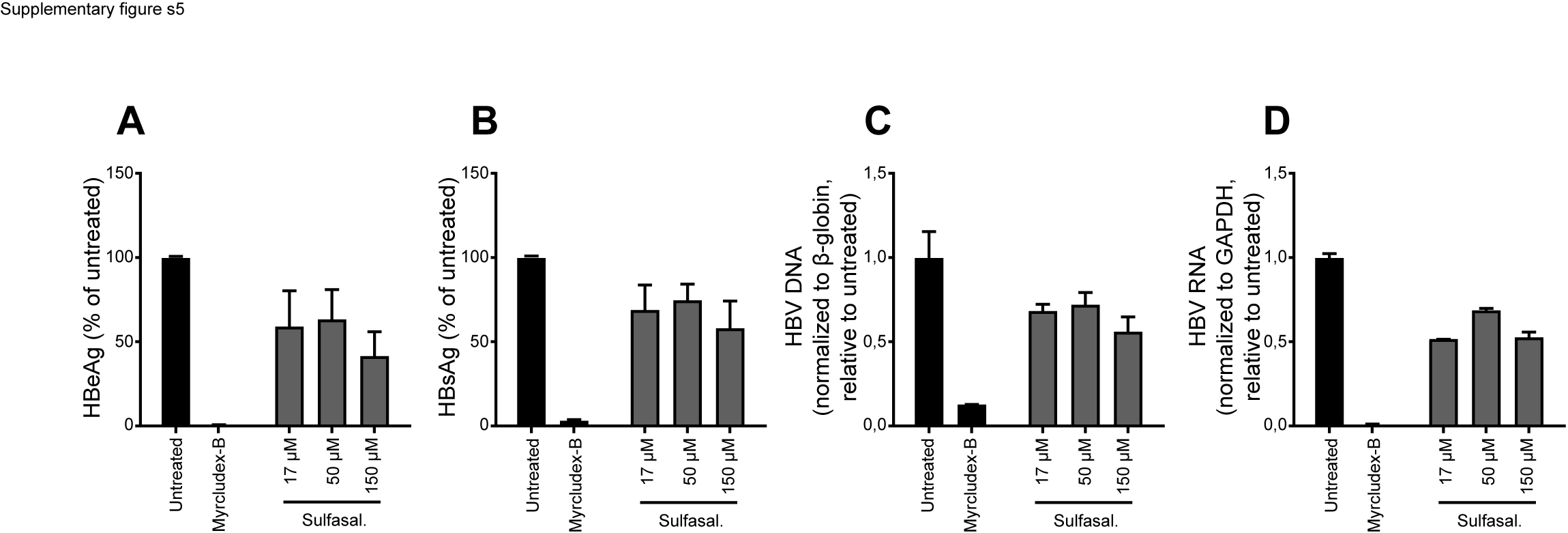
**

**Supplementary Figure S3** **Inhibition of serum-derived HBV in NTCP-overexpressing HepaRg cells using sulfasalazine.** (A-B) Hepatitis B extracellulair (A) and surface (B) antigen production was reduced by sulfasalazine in a dose-dependent fashion. (C-D) Also, sulfasalazine was effective in reducing both HBV DNA (C) and HBV RNA (D). Myrcludex B (1 µM) was included as positive control in all assays. Data are presented as mean ± SD, n= 2-3 wells/condition.


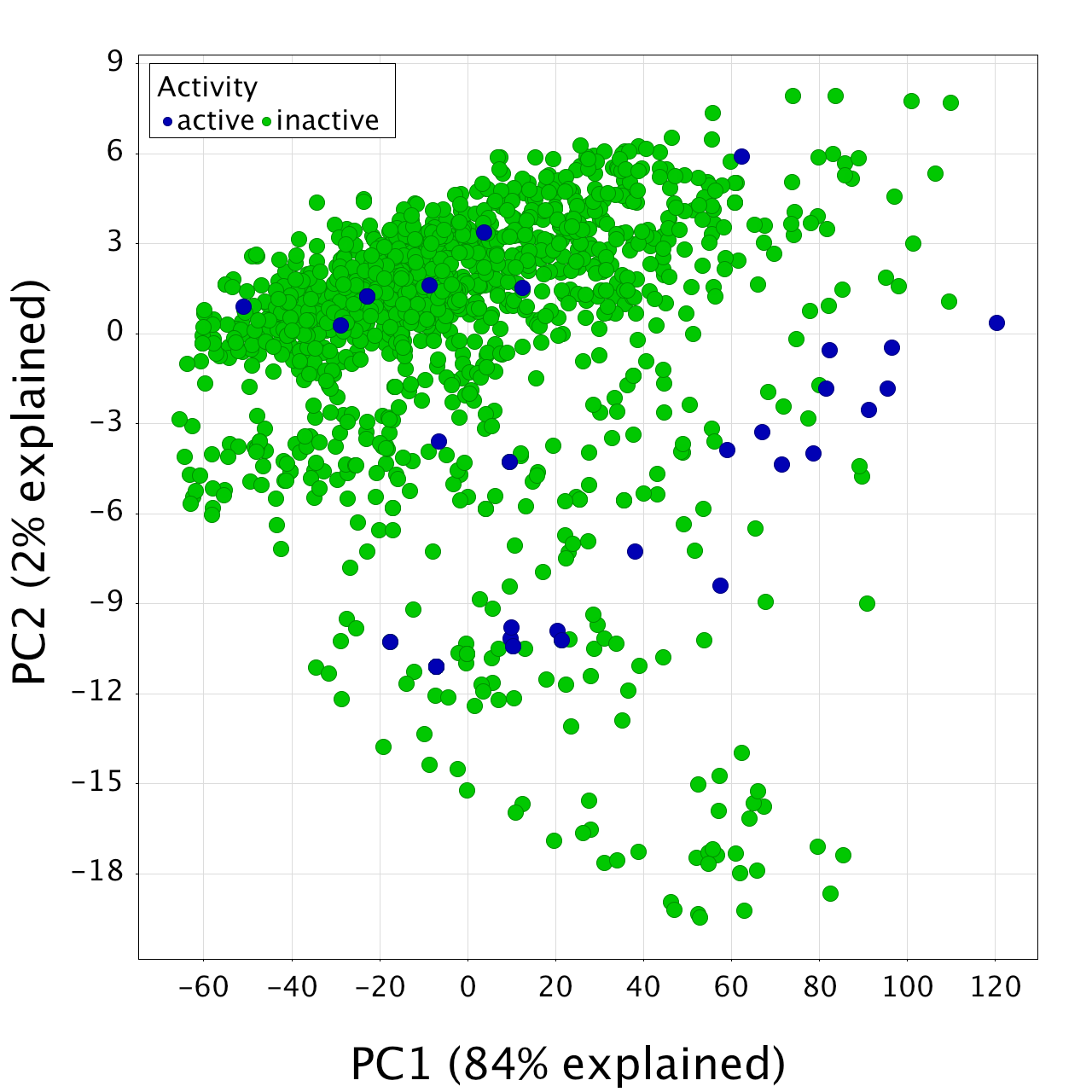

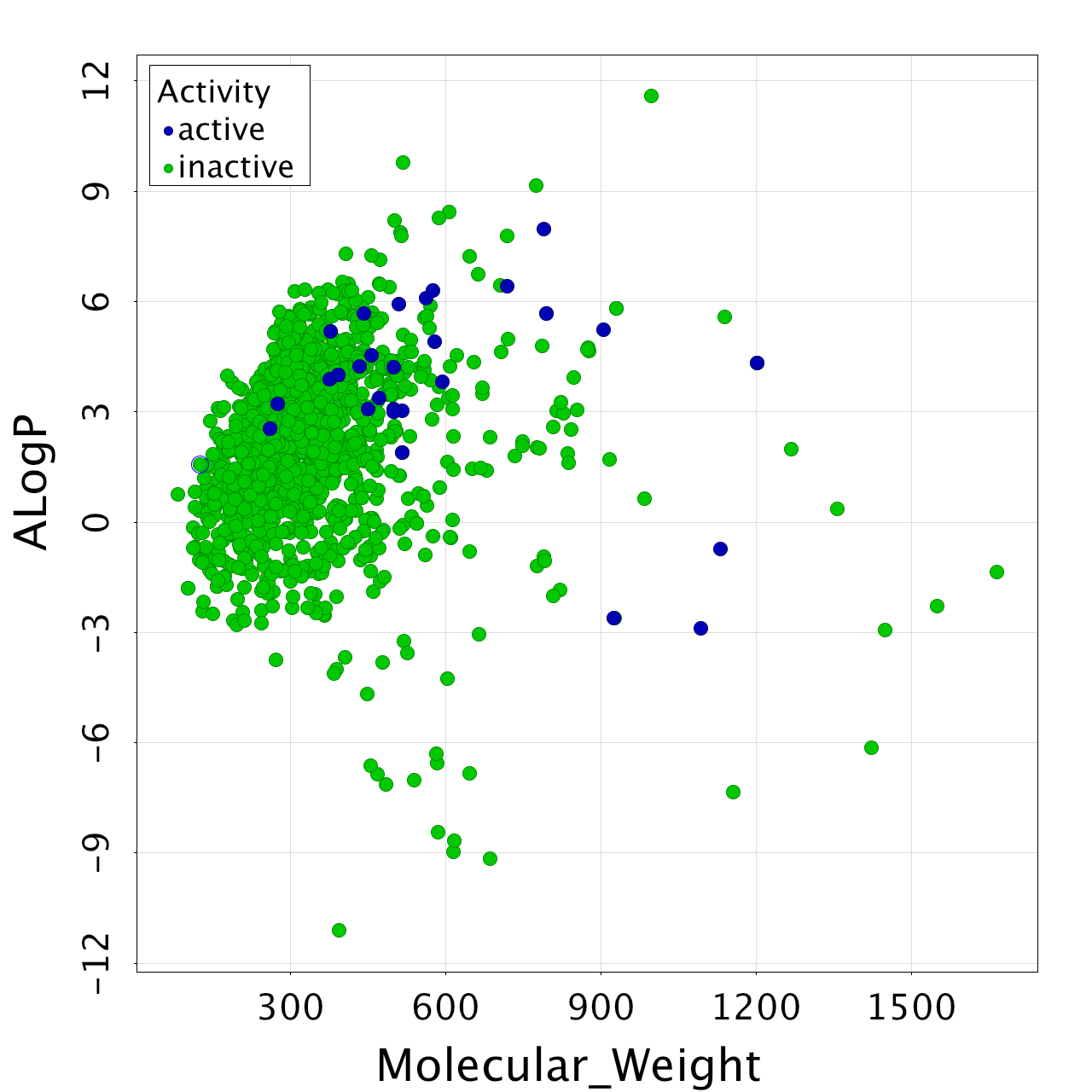

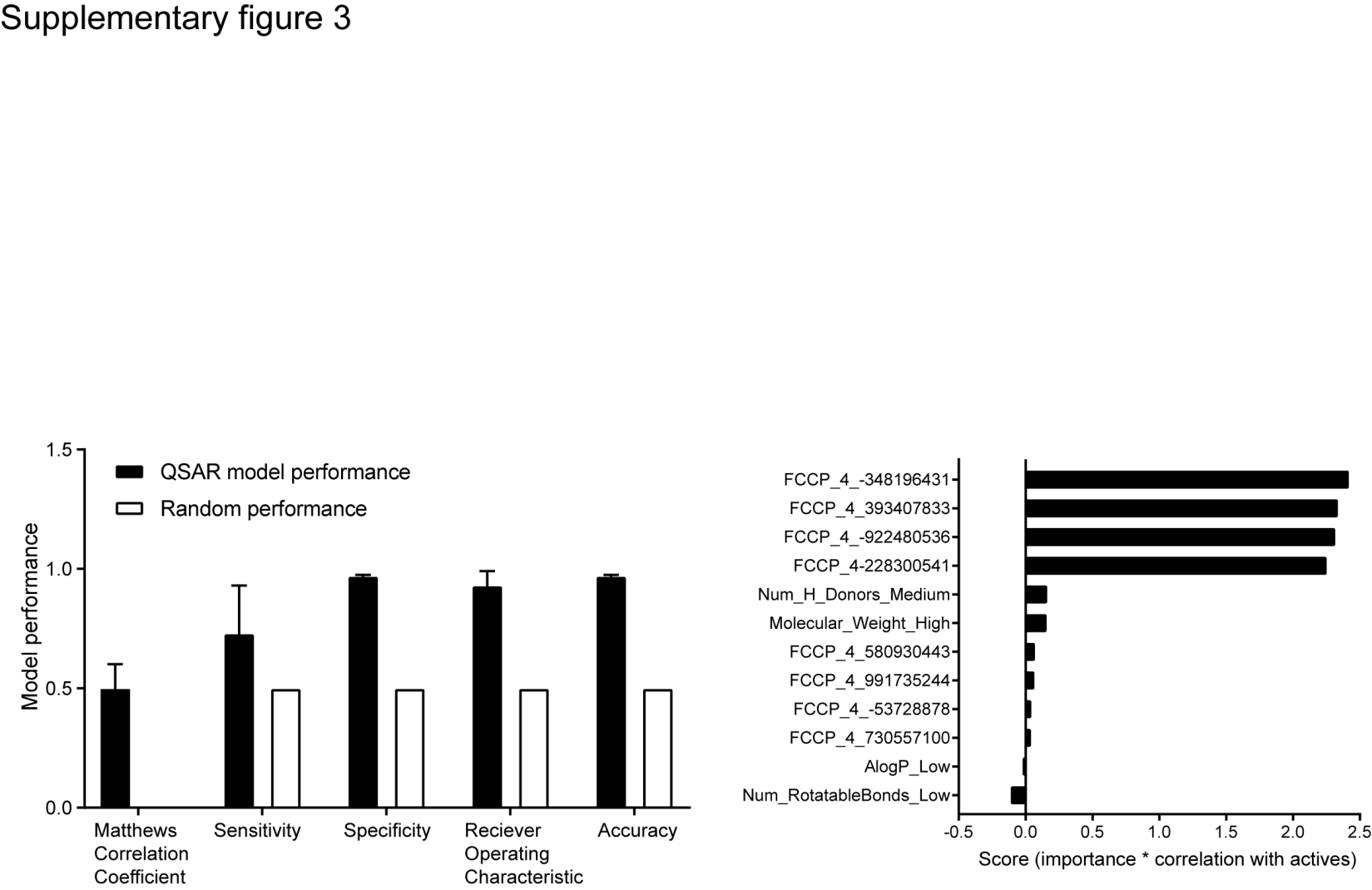


**A**

**B**

**C**

**D**

**Supplementary Figure S4. Cheminformatics analysis.** (A) Scatter plot of drug molecular weight and lipophilicity (AlogP) shows no obvious cluster formation of actives (blue) compared to inactives (green). (B) The same was true for other compounds and a principal component analysis performed on the data. (C) QSAR model performance compared to random performance. The model is predictive with an accuracy of 0.97 (±0.01) and MCC of 0.50 (±0.11). Random performance for accuracy in binary classification is 0.50 and for MCC 0.00. (D) Property importances obtained from the 5 QSAR models. Shown are the top and bottom four most important chemical substructures and the top and bottom two performing physicochemical properties.


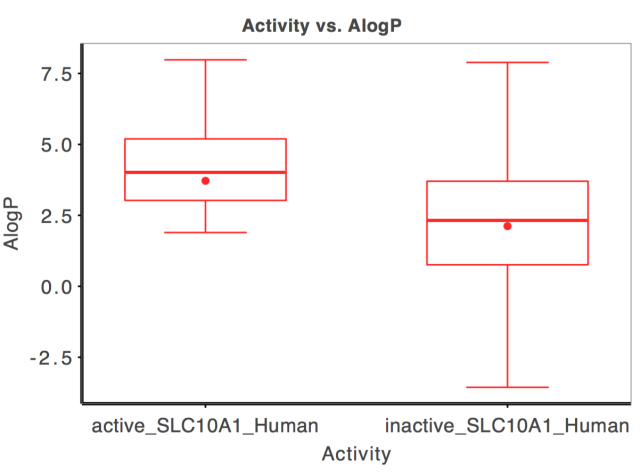

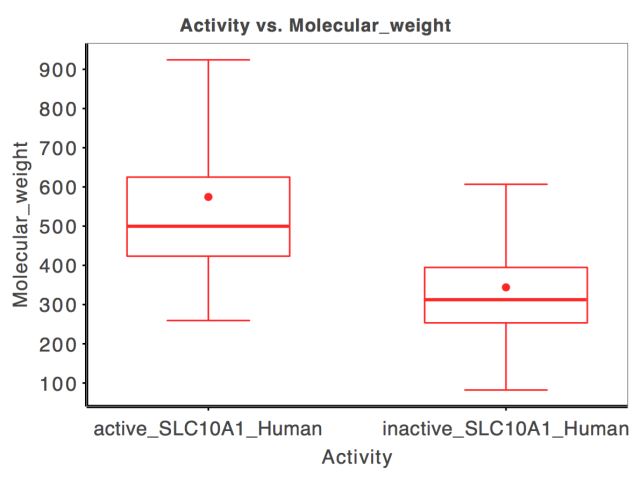

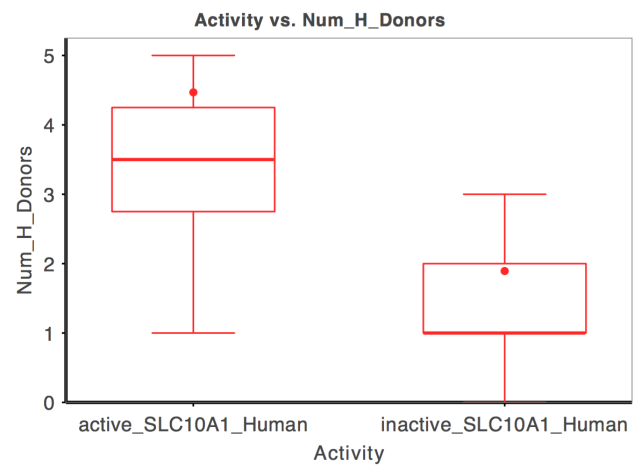

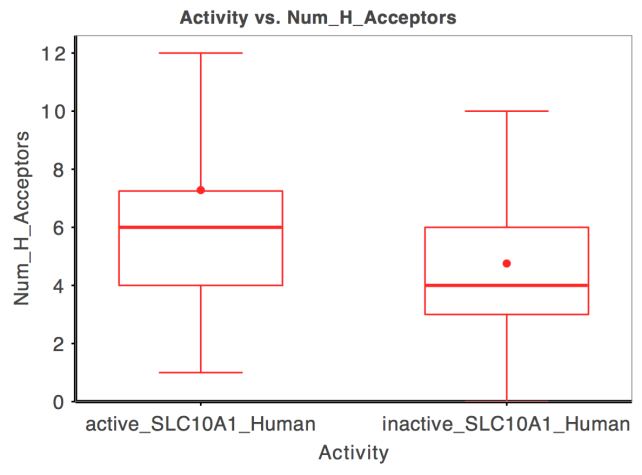

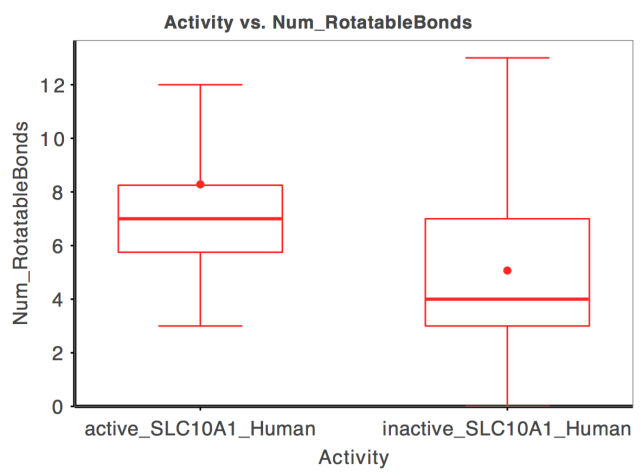

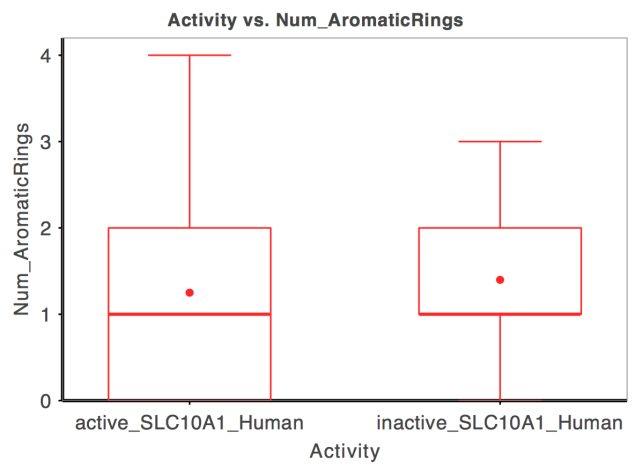

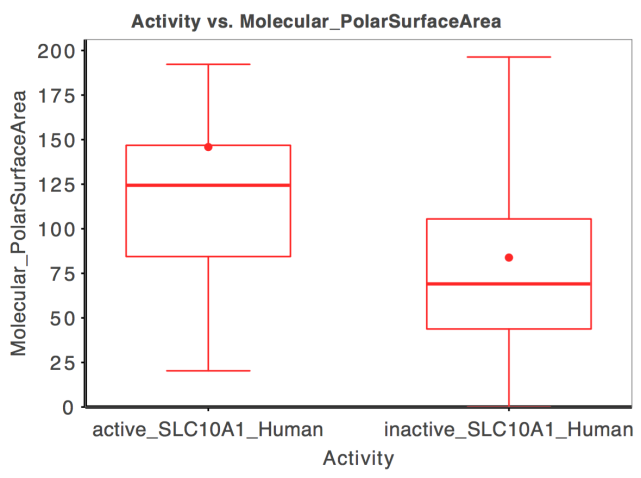


**C**

**A**

**B**

**D**

**E**

**G**

**F**

**Supplementary Figure S5. Property differences between actives and inactives in the cheminformatics dataset.** (A-G) Boxplots representing differences in the cheminformatics dataset between actives and inactives for AlogP (A), molecular weight (B), number of hydrogen bond donors (C) and acceptors (D), rotable bonds (E), aromatic rings (F), and molecular polar surface area (G). The dot indicates the mean value, the bold line the median value. Limits of the box indicate the low and high percentile (25 and 75 respectively), the whiskers indicate the 1.5 interquartile range.

**References**

1 De Bruyn, T. *et al.* Structure-based identification of OATP1B1/3 inhibitors. *Mol Pharmacol* **83**, 1257-1267, doi:10.1124/mol.112.084152 (2013).

2 Rogers, D. & Hahn, M. Extended-connectivity fingerprints. *J Chem Inf Model* **50**, 742-754, doi:10.1021/ci100050t (2010).

3 Breiman, L. Random forests. *Mach Learn* **45**, 5-32, doi:Doi 10.1023/A:1010933404324 (2001).

4 Matthews, B. W. Comparison of Predicted and Observed Secondary Structure of T4 Phage Lysozyme. *Biochim Biophys Acta* **405**, 442-451, doi:Doi 10.1016/0005-2795(75)90109-9 (1975).

5 FDA. *Azulfidine sulfasalazine tablets*, <https://www.accessdata.fda.gov/drugsatfda_docs/label/2009/007073s124lbl.pdf> (2009).

6 FDA. *Avandia (rosiglitazone maleate) tablets*, <https://www.fda.gov/downloads/drugs/drugsafety/postmarketdrugsafetyinformationforpatientsandproviders/ucm143413.pdf> (2007).

7 T3TB. *Tiratricol (T3D4983)*, <http://www.t3db.ca/toxins/T3D4983> (2014).

8 AstraZenecaPharmaceuticals. *Accolate (zafirlukast) tablet*, <https://dailymed.nlm.nih.gov/dailymed/archives/fdaDrugInfo.cfm?archiveid=2948> (2006).
